# Interleukin-6 and Cardiovascular Events in Healthy Adults: MESA

**DOI:** 10.1016/j.jacadv.2024.101063

**Published:** 2024-07-09

**Authors:** Muhammad Shahzeb Khan, Khawaja M. Talha, Muhammad Haisum Maqsood, Jennifer A. Rymer, Barry A. Borlaug, Kieran F. Docherty, Ambarish Pandey, Florian Kahles, Maja Cikes, Carolyn S.P. Lam, Anique Ducharme, Adrian A. Voors, Adrian F. Hernandez, A. Michael Lincoff, Mark C. Petrie, Paul M. Ridker, Marat Fudim

**Affiliations:** a Duke University Medical Center, Duke Clinical Research Institute, Durham, North Carolina, USA; b Department of Medicine, University of Mississippi Medical Center, Jackson, Mississippi, USA; c Department of Cardiology, DeBakey Heart and Vascular Center, Houston Methodist Hospital, Houston, Texas, USA; d Department of Cardiovascular Medicine, Mayo Clinic, Rochester, Minnesota, USA; e Institute of Cardiovascular and Medical Sciences, University of Glasgow, Glasgow, United Kingdom; f Division of Cardiology, Department of Internal Medicine, University of Texas Southwestern Medical Center, Dallas, Texas, USA; g Department of Internal Medicine I-Cardiology, University Hospital Aachen, Aachen, Germany; h University of Zagreb School of Medicine, University Hospital Centre, Zagreb, Croatia; i Duke-NUS Medical School, Singapore, Singapore; j Institut de Cardiologie, de Montréal, Université de Montréal, Montréal, Québec, Canada; k Department of Cardiology, University Medical Center of Groningen, University of Groningen, Groningen, the Netherlands; l Heart, Vascular, and Thoracic Institute, Cleveland Clinic, Cleveland, Ohio, USA; m Center for Cardiovascular Disease Prevention, Division of Preventive Medicine and the Division of Cardiovascular Medicine, Brigham and Women's Hospital, Boston, Massachusetts, USA

**Keywords:** cardiovascular disease, ethnicity, inflammation, interleukin-6, population, race

## Abstract

**Background:**

Elevated interleukin (IL)-6 levels have been linked to adverse outcomes in patients with and without baseline cardiovascular disease (CVD).

**Objectives:**

The purpose of this study was to examine the association between circulating IL-6 levels and CVD events without baseline CVD across racial and ethnic groups.

**Methods:**

We conducted an observational analysis utilizing the MESA (Multi-Ethnic Study of Atherosclerosis), a multicenter, prospective community-based study of CVD at baseline from four racial and ethnic groups. IL-6 levels were measured at the time of enrollment (visit 1) and were divided into 3 terciles. Patient baseline characteristics and outcomes, including all-cause mortality, CV mortality, heart failure, and non-CV mortality, were included. Cox proportional hazard regression models were used to assess associations between IL-6 levels and study outcomes with IL-6 tercile 1 as reference.

**Results:**

Of 6,622 individuals, over half were women (53%) with a median age of 62 (IQR: 53-70) years. Racial and ethnic composition was non-Hispanic White (39%) followed by African American (27%), Hispanic (22%), and Chinese American (12%). Compared to tercile 1, participants with IL-6 tercile 3 had a higher adjusted risk of and all-cause mortality (HR: 1.98 [95% CI: 1.67-2.36]), CV mortality (HR: 1.55 [95% CI: 1.05-2.30]), non-CV mortality (HR: 2.05 [95% CI: 1.65-2.56]), and heart failure (HR: 1.48 [95% CI: 0.99-2.19]). When tested as a continuous variable, higher levels of IL-6 were associated with an increased risk of all individual outcomes. Compared to non-Hispanic White participants, the unadjusted and adjusted risk of all outcomes across all races and ethnicities was similar across all IL-6 terciles.

**Conclusions:**

High levels of circulating IL-6 are associated with worse CV outcomes and increased all-cause mortality consistently across all racial and ethnic groups.

Interleukin (IL)-6 is an immune-mediated, pro-inflammatory cytokine that is elevated in systemic inflammatory states.^1^ IL-6 plays a direct role in activating endothelial monocytes and macrophages, thereby accelerating plaque accumulation and atherosclerosis.^2^^,^^3^ Elevated levels of circulating IL-6 have consistently shown an association with a higher risk of atherosclerotic cardiovascular disease (CVD) events and all-cause mortality in both patients with existing atherosclerotic CVD and apparently healthy individuals, emphasizing the pivotal role of IL-6 as a mediator in incident CVD development.^4^, ^5^, ^6^ As a result, there are ongoing trials into novel therapies aimed at inhibiting circulating IL-6 for the reduction of CVD events, including the ZEUS (Ziltivekimab Cardiovascular Outcomes Study; NCT05021835) and HERMES trials (A Research Study to Look at How Ziltivekimab Works Compared to Placebo in People With Heart Failure and Inflammation; NCT05636176).^7^^,^^8^

There may be differences in baseline levels of IL-6 and strength of association with CVD outcomes across different races and ethnicities. In a cohort of patients with baseline chronic kidney disease, African American individuals had significantly higher levels of circulating IL-6 levels compared to Mexican American and non-Hispanic (NH) White individuals.^9^ Barrow et al also found significantly higher IL-6 levels in Black adults compared to White adults; however, the association between circulating IL-6 and CV mortality was stronger among White adults compared to Black adults.^10^ Similar evidence for the association of IL-6 levels with CVD outcomes in individuals without clinical CVD is less well-established. Cainzos-Achirica et al evaluated the prognostic implications of IL-6 levels on hard coronary heart disease (CHD) events, stroke, hard atherosclerotic CVD, and incident heart failure (HF) in patients without clinical CVD using the MESA (Multi-Ethnic Study of Atherosclerosis); however, the analysis was focused on assessing differences between users and nonusers of statins and did not assess the impact of race and ethnicity on clinical outcomes.^11^ It is pertinent to assess outcomes in the context of differences in circulating IL-6 across races and ethnicities to better understand overall population-level risk of CVD and identify high-risk cohorts that would potentially benefit from anti-IL-6 therapies. In this study, we utilized patient-level data from the MESA cohort to examine the association between circulating IL-6 levels and the occurrence of CVD events in individuals without clinical CVD at baseline and evaluated for the presence of effect modification across different racial and ethnic groups.

## Methods

### Study design

The MESA is a multicenter, National Heart, Lung, and Blood Institute-funded, community-based cohort study of apparently healthy men and women without clinical CVD (eg, myocardial infarction, stroke, peripheral artery disease, HF, chronic kidney disease) at baseline from four racial and ethnic groups recruited in 6 sites within the United States. The details of the MESA study have been reported elsewhere^12^ and also available on the study website (https://www.mesa-nhlbi.org). The MESA study was approved by the respective Institutional Review Boards at each participating site, and individual informed consent was taken from all participants.

### Study population

All participants evaluated in MESA visit 1 were included (N = 6,814). Of 6,814 participants, those with missing information or unmeasured IL-6 were excluded. Finally, 6,622 patients with no CVD at baseline were included.

### Event ascertainment

Patient characteristics obtained at baseline were recorded. Participants were contacted every 9 to 12 months to inquire about CVD outpatient diagnoses, hospital admissions, and procedures. CV mortality was defined as death due any atherosclerotic disease, stroke, CHD, and other CVD. Deaths not attributed to aforementioned were considered as non-CV mortality. HF was defined by the presence of symptoms suggestive of the disease (eg, shortness of breath, edema), a clinician diagnosis, and use of medical therapy for HF.

### IL-6 measurement

Levels of IL-6 were measured in visit 1 from blood samples, using an ultrasensitive enzyme-linked immunosorbent assay (Quantikine HS Human IL-6 Immunoassay; R&D Systems) and quantified in pg/mL. The analytical coefficient of variation was 6.3% (detection level 0.04 pg/mL). N-Terminal pro-B natriuretic peptide and cardiac troponin T were measured in serum collected at the baseline exam 1 using the Elecsys 2010 system (Roche Diagnostics).

### Statistical analysis

Categorical variables were reported as frequency and percentage while continuous variables (including IL-6) were reported as median (IQR). IL-6 was also categorically divided into terciles (named as tercile 1, tercile 2, and tercile 3). Tercile 1 indicated the lower one-third of IL-6 values while tercile 3 indicated the upper one-third IL-6 values. Unadjusted incidence rates per 1,000 person-years and unadjusted Kaplan-Meier cumulative survivor function curves were calculated by categories of IL-6 terciles for each of the study end points. Cox proportional hazards regression models were used to assess associations between IL-6 and each of the study outcomes with IL-6 tercile 1 as a reference using 2 models. Model 1 was crude (unadjusted), whereas model 2 was adjusted for age at baseline, sex, race/ethnicity, current smoking, family history of CHD, waist circumference, systolic blood pressure, total cholesterol, high-density lipoprotein cholesterol, low-density lipoprotein cholesterol, diabetes, hypertension, aspirin use, antihypertensive medication use, insulin use, statin use, cardiac troponin T levels, and serum N-Terminal pro-B natriuretic peptide (all assessed at baseline visit 1). This was performed to account for the effect of sociodemographic and clinical variables on circulating IL-6 levels and outcomes. Using Cox proportional hazards models, we assumed that survival curves for different strata must have hazard functions that are proportional over the time. Test of Cox proportional hazards assumptions was assessed using a global test with *P* value <0.05 implying that survival curves for different strata have hazard functions that are not proportional over the time. A *P* value of <0.05 was considered the threshold for statistical significance. All analyses were performed using Stata software, version 18 (StataCorp LLC).

### Subgroups analyses

We performed a subgroup analysis to study the association between circulating IL-6 levels and CVD outcomes across races and ethnicities. Crude incident rates per 1,000 person-years for NH White adults, African American adults, and Hispanic adults were calculated by categories of IL-6 terciles for each outcome. Cox proportional hazard regression models, similar to those described above, were used to assess the association between IL-6 and study end points with IL-6 tercile 1 as reference for Model 1 (unadjusted) and Model 2 (fully adjusted as described earlier). A competing risk analysis was performed for incident HF, and a cumulative incidence analysis using competing risk regression was performed across IL-6 terciles for incident HF. Interaction analyses were performed to evaluate the risk of CVD events in African American and Hispanic participants compared to the risk in NH Whites (set as reference). *P* value for interaction of <0.05 was considered significant.

## Results

A total of 6,622 individuals without known CVD at baseline were evaluated, with a median follow-up duration of 14 years (IQR: 12.21-14.65 years). The cohort was divided into 3 terciles based on circulating IL-6 levels obtained at enrollment: tercile 1: 2,208 (0.65 pg/mL [95% CI: 0.51-0.78 pg/mL]), tercile 2: 2,207 (1.21 pg/mL [95% CI: 1.04-1.38 pg/mL]), and tercile 3: 2,207 (2.23 pg/mL [95% CI: 1.89-3.24 pg/mL]). The median circulating IL-6 levels across the total cohort was 1.21 (IQR: 0.78-1.89) pg/mL. The participants were predominantly female (52.9%) and had a median age of 62 (IQR: 53-70) years, with older age and higher proportion of female sex at higher IL-6 terciles. Majority of the patients were NH White (38.8%) followed by African Americans (27.3%), Hispanic (22.0%), and Chinese Americans (12.0%) (Table 1).

**Table 1:** Baseline Characteristics of Patients With IL-6 Levels Divided Into 3 Terciles

	IL-6 Terciles			All Patients	*P* Value
	Tercile 1 (n = 2,208)	Tercile 2 (n = 2,207)	Tercile 3 (n = 2,207)		
IL-6, pg/mL	0.65 (0.51-0.78)	1.21 (1.04-1.38)	2.32 (1.89-3.24)	1.21 (0.78-1.89)	<0.001
Age, y	58 (51-67)	63 (54-71)	65 (55-72)	62 (53-70)	<0.001
Male	1,134 (51.4)	1,031 (46.7)	954 (43.2)	3,119 (47.1)	<0.001
Race					<0.001
Non-Hispanic White	946 (42.8)	875 (39.6)	747 (33.8)	2,568 (38.8)	
Chinese American	429 (19.4)	226 (10.2)	139 (6.3)	794 (12.0)	
African American	471 (21.3)	609 (27.6)	725 (32.9)	1,805 (27.3)	
Hispanic	362 (16.4)	497 (22.5)	596 (27.0)	1,455 (22.0)	
Social history					
Current smoker	226 (10.2)	271 (12.3)	353 (16.0)	850 (12.8)	<0.001
Family history of CHD	822 (39.4)	924 (44.6)	903 (44.0)	2,649 (42.6)	<0.001
Physiologic measures					
SBP, mm Hg	118.5 (107-133.5)	125 (112-140.5)	127.5 (114.5-143)	123.5 (111-139.5)	<0.001
Heart rate, beats/min	61 (55-67)	62 (57-69)	64 (58-71)	62 (56-69)	<0.001
Waist circumference, cm	91.1 (83.5-99.3)	97.8 (90-106.4)	103.1 (94.2-113.5)	97.1 (88.2-106.7)	<0.001
BMI, kg/m^2^	25.6 (23.1-28.3)	27.9 (25.0-31.0)	29.9 (26.4-34.3)	27.6 (24.6-31.1)	<0.001
Laboratory investigations					
Creatinine, mg/dL	0.9 (0.80-1,1)	0.9 (0.8-1.1)	0.9 (0.8-1.1)	0.9 (0.8-1.1)	0.47
Total cholesterol, mg/dL	194 (173-217)	195 (173-218)	188 (166-212)	192 (171-215)	<0.001
HDL cholesterol, mg/dL	51 (42-62)	48 (40-59)	46 (39-56)	48 (40-59)	<0.001
LDL cholesterol, mg/dL	116 (97-137)	118 (98-138)	113 (93-133)	116 (96-136)	<0.001
Triglyceride, mg/dL	102 (72-147)	117 (80-169)	116 (83-168)	112 (78-161)	<0.001
Troponin T, ng/mL	0.009 (0.009-0.009)	0.009 (0.009-0.009)	0.009 (0.009-0.009)	0.009 (0.009-0.009)	<0.001
NT-proBNP, pg/mL	45.0 (20.7-88.1)	55.8 (23.5-111.7)	65.1 (30.1-140.4)	54.4 (24.0-111.8)	<0.001
Medical history					
Hypertension	721 (32.7)	1,053 (47.7)	1,182 (53.6)	2,956 (44.6)	<0.001
Diabetes	157 (7.1)	252 (11.4)	340 (15.4)	749 (11.3)	<0.001
Medication use					
Antihypertensive medications	589 (26.7)	857 (38.8)	1,002 (45.4)	2,448 (37.0)	<0.001
Aspirin use	515 (23.3)	570 (25.8)	570 (25.8)	1,655 (25.0)	0.086
Insulin use	16 (0.70)	32 (1.5)	69 (3.1)	117 (1.8)	<0.001
Statin use	308 (14.0)	353 (16.0)	319 (14.5)	980 (14.8)	0.14
Any lipid-lowering agent	331 (15.0)	383 (17.4)	355 (16.1)	1,069 (16.2)	0.10

Values are median (IQR) or n (%).

BMI = body mass index; CHD = coronary heart disease; HDL = high-density lipoproteins; IL-6 = interleukin-6; LDL = low-density lipoproteins; NT-proBNP = N-terminal pro-B natriuretic peptide; SBP = systolic blood pressure.

### Baseline variables

A minority of participants were current smokers (12.8%), 42.6% had a family history of CHD, 44.6% had hypertension, and 11.3% had diabetes. A higher proportion of patients in higher IL-6 terciles were current smokers, had a family history of CHD, had diabetes, and had hypertension at baseline (*P* < 0.001 for all). The median serum creatinine was 0.9 (IQR: 0.8-1.1) mg/dL with no significant trend across IL-6 tertiles (*P* = 0.47). The total cholesterol, high-density lipoprotein, low-density lipoprotein, and triglyceride levels were 192 (IQR: 171-215) mg/dL, 48 (IQR: 40-59) mg/dL, 116 (IQR: 96-136) mg/dL, and 112 (IQR: 78-161 mg/dL), respectively. Baseline use of antihypertensive agents was most common (37.0%), followed by aspirin (25.0%), any lipid-lowering agent (16.2%), statins (14.8%), and insulin (1.8%) (Table 1).

### CV outcomes

#### All-cause mortality

**Figure 1:**
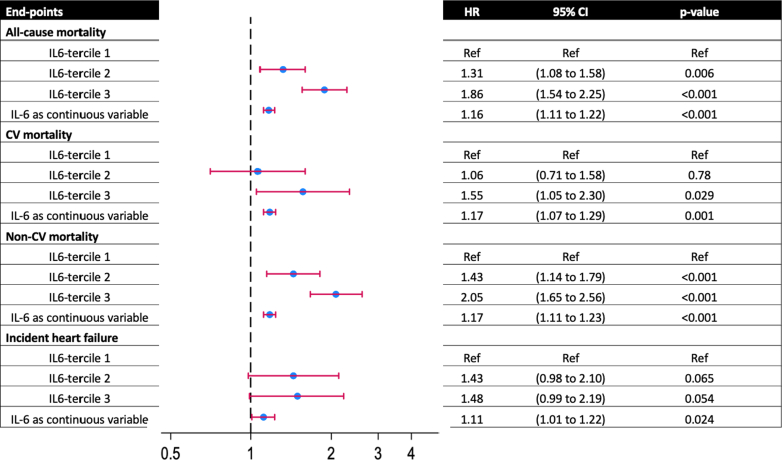
**Cox Proportional Hazard Models Comparing the Risk of Cardiovascular Outcomes Across IL-6 Terciles** Tercile 1 is used as reference to compare outcomes with patients in IL-6 terciles 2 and 3. IL-6 was also analyzed as a continuous variable for each outcome of interest. CV = cardiovascular; IL = interleukin.

**Figure 2:**
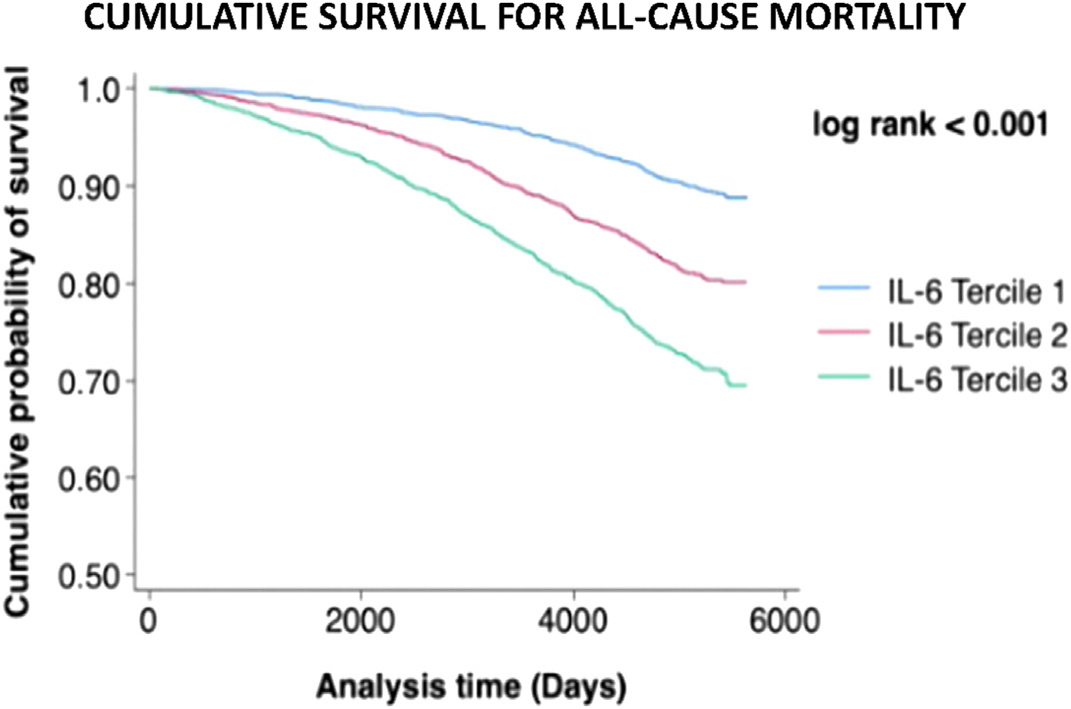
**Kaplan-Meier Estimates for Patients With IL-6 Levels in Terciles 1 to 3 for All-Cause Mortality** IL = interleukin.

The unadjusted overall incidence of all-cause mortality was 14.5 per 1,000 (IQR: 13.7-15.3) person-years. Individuals in IL-6 tercile 3 had a higher risk of all-cause mortality compared to individuals in tercile 1 (adjusted HR: 1.98 [95% CI: 1.67-2.36]). When tested as a continuous variable, higher IL-6 levels were associated with a higher risk of all-cause mortality in both unadjusted and fully adjusted models (Supplemental Table 1, Figure 1). Crude Kaplan-Meier survival estimates indicate significantly lower cumulative probability of survival with early divergence of survival curves for IL-6 terciles 2 and 3, compared to tercile 1 (log rank *P* < 0.001) (Figure 2).

#### CV mortality

The unadjusted overall incidence of CV mortality was 3.79 per 1,000 person-years (IQR: 3.39-4.25). Individuals in IL-6 terciles 3 had a higher risk of CV mortality compared to individuals in IL-6 tercile 1 (adjusted HR: 1.55 [95% CI: 1.05-2.30]). When tested as a continuous variable, higher IL-6 levels were associated with a higher risk of CV mortality in both unadjusted and fully adjusted models (Supplemental Table 1, Figure 1).

#### Non-CV mortality

The unadjusted overall incidence of non-CV mortality was 10.9 per 1,000 (IQR: 10.2-11.7) person-years. Individuals in IL-6 tercile 3 had a significantly higher risk of non-CV mortality compared to individuals in IL-6 tercile 1 (adjusted HR: 2.05 [95% CI: 1.65-2.56]). When tested as a continuous variable, higher IL-6 levels were associated with a higher risk of non-CV mortality in both unadjusted and fully adjusted models (Supplemental Table 1, Figure 1).

#### Incident HF

**Figure 3:**
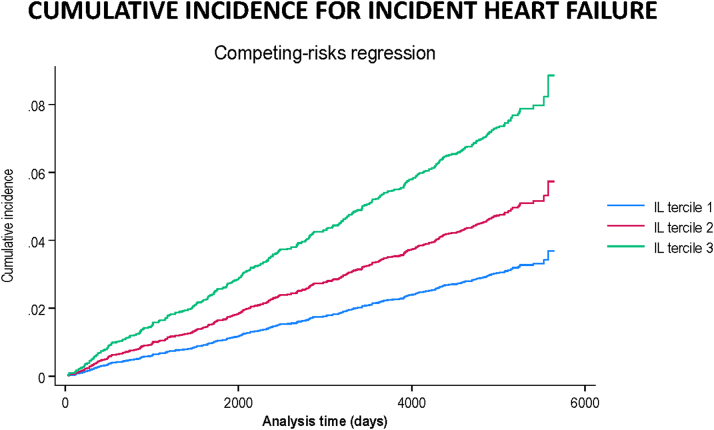
**Cumulative Incidence Estimates for Patients With IL-6 Levels in Terciles 1 to 3 for Incident Heart Failure** IL = interleukin.

The unadjusted overall incidence of incident HF was 4.09 per 1,000 (IQR: 3.67-4.55) person-years. Individuals in IL-6 tercile 3 had a similar risk of incident HF compared to individuals in tercile 1 (adjusted HR: 0.80 [95% CI: 0.45-1.45]). When tested as a continuous variable, the risk of HF with CVD did not increase with increasing IL-6 levels (Supplemental Table 1, Figure 1). A competing risk analysis using all-cause mortality yielded similar associations (Supplemental Table 2). A cumulative incidence analysis for incident HF across the IL-6 terciles using competing risk regression is provided in Figure 3.

#### Outcomes stratified by race and ethnicity

African American and Hispanic participants constituted a higher proportion of total patients in higher IL-6 terciles compared to lower terciles (African American: 21.3% in tercile 1 vs 32.9% in tercile 3; Hispanic: 16.4% in tercile 1 vs 27.0% in tercile 3), whereas the proportion of NH White and Chinese American participants decreased with increasing IL-6 terciles (NH White: 42.8% in tercile 1-33.8% in tercile 3; Chinese American: 19.4% in tercile 1 vs 6.1 in tercile 3) (Table 1). Table 2 illustrates the interaction analysis between racial and ethnic groups and outcomes across IL-6 terciles. The adjusted risk of CV mortality was similar for African American and Hispanic participants compared to NH White participants across all IL-6 terciles (*P* > 0.05 for all). Non-CV mortality was similar among African Americans and NH White participants across all IL-6 terciles, whereas Hispanic participants had a lower risk of non-CV mortality in tercile 1 and 2 on unadjusted analysis with no statistical significance in tercile 1 on adjusted analysis (*P* = 0.14). The risk of overall HF was not significantly different among African American and Hispanic participants compared to NH White participants across all IL-6 terciles on both unadjusted and fully adjusted analyses. Additionally, the risk of all-cause mortality was similar among African American and Hispanic participants compared to NH White participants on both unadjusted and fully adjusted analyses.

**Table 2:** Cox Proportional Hazard Model and *P* Value for Interaction in African American and Hispanics Adults With Reference to Non-Hispanic Whites

Tercile Groups	African American				Hispanics			
	HR (95% CI)	***P*Value for** Interaction With NHW	HR (95% CI)	***P*Value for** Interaction With NHW	HR (95% CI)	***P*Value for** Interaction With NHW	HR (95% CI)	***P*Value for** Interaction With NHW
	Model 1^a^		Model 2^b^		Model 1^a^		Model 2^b^	
All-cause mortality								
IL-6 tercile 1	0.88 (0.62-1.25)	0.49	0.85 (0.51-1.41)	0.54	0.52 (0.33-0.84)	0.007	0.75 (0.45-1.26)	0.27
IL-6 tercile 2	1.17 (0.93-1.46)	0.18	1.22 (0.83-1.80)	0.31	0.78 (0.59-1.02)	0.07	0.87 (0.63-1.22)	0.43
IL-6 tercile 3	1.04 (0.86-1.25)	0.72	1.22 (0.90-1.67)	0.21	0.85 (0.69-1.05)	0.13	0.98 (0.76-1.26)	0.88
CV mortality								
IL-6 tercile 1	0.84 (0.39-1.82)	0.66	0.55 (0.19-1.57)	0.26	0.82 (0.35-1.92)	0.65	1.04 (0.41-2.62)	0.93
IL-6 tercile 2	1.32 (0.82-2.11)	0.26	0.88 (0.41-1.90)	0.75	1.00 (0.58-1.70)	0.99	0.85 (0.44-1.63)	0.62
IL-6 tercile 3	1.43 (0.97-2.11)	0.074	0.85 (0.49-1.48)	0.57	0.86 (0.54-1.36)	0.52	0.64 (0.37-1.09)	0.10
Non-CV mortality								
IL-6 tercile 1	0.92 (0.62-1.36)	0.67	0.94 (0.51-1.72)	0.84	0.43 (0.24-0.77)	0.005	0.62 (0.32-1.20)	0.16
IL-6 tercile 2	1.10 (0.85-1.44)	0.47	1.29 (0.80-2.08)	0.30	0.66 (0.48-0.92)	0.014	0.80 (0.53-1.20)	0.29
IL-6 tercile 3	0.94 (0.75-1.18)	0.60	1.36 (0.91-2.03)	0.13	0.83 (0.66-1.06)	0.14	1.09 (0.81-1.46)	0.57
Incident heart failure								
IL-6 tercile 1	1.18 (0.64-2.18)	0.59	1.15 (0.43-3.12)	0.78	0.37 (0.13-1.06)	0.064	0.52 (0.15-1.81)	0.30
IL-6 tercile 2	0.91 (0.58-1.42)	0.68	0.78 (0.34-1.81)	0.56	1.01 (0.65-1.60)	0.95	0.87 (0.47-1.62)	0.66
IL-6 tercile 3	1.09 (0.75-1.60)	0.65	0.97 (0.48-1.97)	0.93	0.93 (0.61-1.41)	0.73	0.82 (0.48-1.41)	0.66

CV = cardiovascular; IL-6 = interleukin-6; NHW = non-Hispanic White.

a Model 1 was crude (unadjusted).

b Model 2 adjusted for age at baseline, sex, race/ethnicity, current smoking, family history of CHD, waist circumference, systolic blood pressure, total cholesterol, HDL cholesterol, LDL cholesterol, diabetes, hypertension, aspirin use, antihypertensive medication use, insulin use, statin use, troponin T, and NT-proBNP (all assessed at baseline).

## Discussion

The study reveals several key findings. Patients with higher IL-6 levels were more likely to be older, female, current smokers; had hypertension, diabetes, and a family history of CHD; had a higher median systolic blood pressure, heart rate, and waist circumference; and included a larger proportion of African American and Hispanic individuals compared to those with lower IL-6 levels. Consistent with prior meta-analyses,^13^ in this multiethnic cohort, elevated IL-6 levels were consistently associated with a higher incidence of all major CVD outcomes even after adjustment for a broad range of clinical covariates, with no major difference in outcomes across race and ethnicities (Central Illustration).

**Central Illustration:**
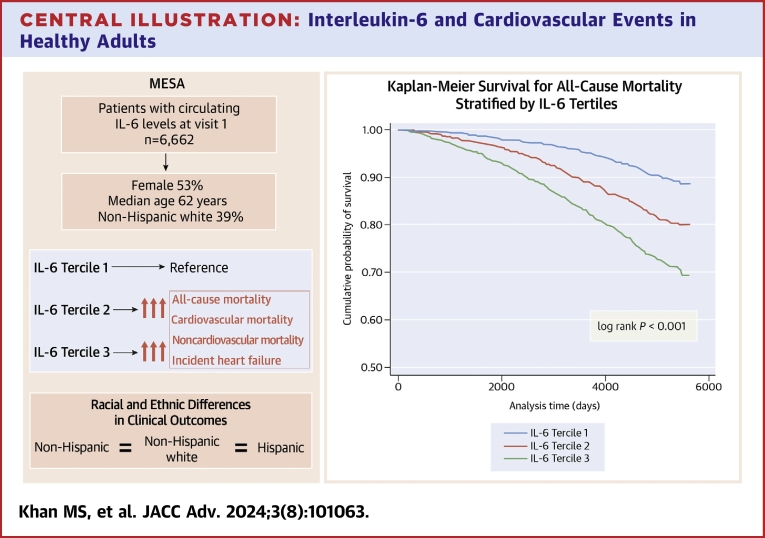
**Interleukin-6 and Cardiovascular Events in Healthy Adults** Diagrammatic overview of the baseline characteristics, association of overall clinical outcomes with il-6 terciles, differences across races and ethnicities, and kaplan-meier estimates for all-cause mortality for patients with IL-6 levels in terciles 1 to 3. IL = interleukin.

These findings from a prospective cohort of closely followed patients with average baseline risk of CVD further strengthen the prognostic role of circulating IL-6 in mediating atherosclerotic CVD risk. A prior analysis from the MESA cohort reported similar findings over a follow-up of 10 years and focused on individual components of atherosclerotic CVD and all-cause death stratified by user and nonusers of statins, whereas our study additionally evaluated association between IL-6 and non-CVD events including mortality and HF.^11^ Evidence from genetic studies has demonstrated a potentially causal role of IL-6 in the development and the acceleration of atherosclerosis.^3^^,^^14^ IL-6 signaling is also implicated in coronary and peripheral arterial disease, and formation of aortic aneurysm, independent of established risk factors suggesting significant direct effects on systemic arterial vasculature associated with circulating IL-6.^6^^,^^15^^,^^16^ Ridker et al previously reported that healthy men with elevated baseline IL-6 levels had a higher risk of development of myocardial infarction compared to an age and smoking status matched cohort even after adjustment for other CVD risk factors.^6^ In further prospective nested case-control analysis, the same group found baseline circulating C-reactive protein (CRP) and IL-6 levels to be a strong predictor of adverse CVD events in healthy men, and circulating CRP, which is considered a surrogate to circulating IL-6 levels,^17^ in healthy women.^18^^,^^19^

The association between elevated IL-6 levels and incident HF is another important finding of our study. Prior analysis from the MESA cohort revealed that high circulating IL-6 levels are related to a reduced regional left ventricular systolic function in apparently healthy individuals^20^ and are associated with increased risk of incident HF.^11^ Studies have consistently reported an increased risk of incident HF in patients with high IL-6 levels, with the association mostly attributed to HF with reduced ejection fraction,^21^ although some studies have reported a higher risk of HF with preserved ejection fraction.^22^^,^^23^ In patients with prevalent HF and preserved ejection fraction, those with increased IL-6 display greater body fat, particularly visceral fat,^24^ and the present data extend this relationship to patients without apparent CVD in MESA.

We also found a significant association between high circulating IL-6 levels and all-cause mortality in a healthy, relatively young cohort (median age ∼62 years). This indicates that a pro-inflammatory state even in younger adults with average CVD risk leads to worse outcomes. Previous studies have shown a similar association of IL-6 levels in elderly patients with established CVD, a patient cohort with known risk factors for a pro-inflammatory state (aging, coronary artery disease, etc).^4^^,^^25^ Lindmark et al found an increased risk of all-cause mortality in patients with elevated IL-6 levels and unstable coronary artery disease and reported a mortality benefit with early invasive management in those with high IL-6 levels.^5^ It can be postulated that elevated IL-6 levels in healthy adults can allow for identification of a high-risk cohort that may benefit from more aggressive preventive interventions.

This analysis was conducted in a large group of racially and ethnically diverse population with a high representation of African American participants (27%) and Hispanic participants (22%) in this study. We found that there was an increasing proportion of Hispanic and African American adults from IL-6 terciles 1 to 3, with both racial and ethnic groups constituting 60% of the tercile 3 group, and a corresponding decrease in NH White adults and Chinese American adults.^9^^,^^26^ Prior evidence suggests that the differential levels of IL-6, especially in African American individuals, might be partly explained by higher body mass index which is independently correlated with circulating IL-6 levels.^9^ Current analysis also demonstrated higher body mass index and waist circumference in patients with higher circulating IL-6 levels (Table 1). The unadjusted and adjusted risk of all CV outcomes across all races and ethnicities was similar across all IL-6 terciles. These data suggest that adults from all races and ethnicities with elevated circulating IL-6 levels are at higher risk of most CVD events, despite some racial and ethnic groups (African American and Hispanic participants) having higher circulating IL-6 levels. Prior studies have not specifically explored these associations. Jia et al assessed the association of IL-6 and IL-18 and development of CVD events in the ARIC (Atherosclerosis Risk In Communities) cohort, which is also a racially and ethnically diverse prospective cohort and found that IL-6 levels were related to higher CVD event rate independent of elevation other inflammatory markers like high-sensitivity troponin, natriuretic peptides, and CRP.^27^

These findings confirm that inflammatory markers like IL-6 may have utility in predicting risk of CVD events in average-risk populations and are a potential target for therapeutic agents to prevent CVD events. There is no current clinical consensus or guidance on the routine use of IL-6 testing for risk stratification and prognostication for any age group. Most studies have been performed in middle age to elderly patients where elevated circulating IL-6 levels have positively correlated with an increased risk of worse outcomes. There has also been a growing interest in anti-inflammatory therapies to reduce the risk of adverse CVD events in patients with established CVD. Low-dose colchicine, a drug that inhibits inflammatory pathways upstream of IL-6 in the cytokine cascade, was found to reduce the risk of adverse CVD events in patients with coronary artery disease^28^^,^^29^ and has been approved for secondary prevention among patients with atherosclerotic disease.^30^ In the CANTOS (Canakinumab Anti-Inflammatory Thrombosis Outcomes Study) of IL-1 inhibition in patients with prior myocardial infarction, patients with more robust reductions in IL-6 had greater CV benefit from targeted anti-inflammatory therapy.^31^ This has been followed by further development of the IL-6 ligand inhibitor, ziltivekimab, which is currently being evaluated for efficacy in patients with CVD, chronic kidney disease as well as HF with preserved ejection fraction with elevated CRP.^7^, ^8^, ^9^ Therefore, the finding of a strong association between CVD outcomes and IL-6 levels across all races and ethnicities in this study further underscores the importance of the development of targeted therapies to improve outcomes across a large group of patients with CVD.

### Study Limitations

This study has certain limitations. The MESA registry only recorded circulating IL-6 levels at enrollment, so our analysis does not account for longitudinal variations in circulating IL-6 levels. Moreover, causal inference cannot be inferred given the observational nature of this study. The number of individual events is relatively small in each tercile and may have limited the power to detect significant differences between groups for CVD events. Similarly, number of events when stratified by race and ethnicity are low which may have underpowered the analysis. We adjusted for highly prognostic variables to assess an independent relationship between IL-6 levels and CVD outcomes; however, there may be other unmeasured confounders that were not accounted for. Levels of other biomarkers, for example, CRP, fibrinogen, and IL-1 that closely track with IL-6 levels were not reported which may have provided further insights into the role of inflammation on CVD outcomes in healthy adults and would have provided surrogate evidence to reinforce the role of IL-6 on CVD outcomes. The proportion of patients with reduced or preserved ejection fraction in patients with incident HF was not recorded.

## Conclusions

High levels of circulating IL-6 were found to be associated with worse CVD outcomes and all-cause mortality in this ethnically diverse cohort. This association was consistent for most outcomes across all races and ethnicities. These data warrant further evaluation to understand how therapies can reduce IL-6 through various mechanisms to decrease the risk of CVD and HF. There is also a compelling need to target therapies to reduce circulating levels of pro-inflammatory cytokines like IL-6 that are implicated in CVD.

PERSPECTIVES **COMPETENCY IN MEDICAL KNOWLEDGE:** In this evaluation of the MESA cohort, we examined the association between circulating IL-6 levels and CV events in an ethnically diverse cohort of apparently healthy adults. Participants with higher baseline IL-6 levels had a significantly higher risk of all-cause mortality, CV mortality, non-CV mortality, and HF, with little effect modification across racial and ethnic groups.

**TRANSLATIONAL OUTLOOK:** Elevated levels of circulating IL-6 have consistently shown an association with a higher risk of CV events in apparently healthy individuals. Racial and ethnic differences have historically influenced the population-level prevalence of CV disease; however, these differences do not significantly modify the association between baseline IL-6 levels and risk of future CV events. Further evaluation is warranted to explore IL-6 as a therapeutic target to reduce the risk of CV events in high-risk and healthy individuals.

## Funding support and author disclosures

Dr Rymer has received research grant support from 10.13039/100007560Chiesi Pharmaceuticals, 10.13039/501100016198Idorsia Pharmaceuticals, and the 10.13039/100000968American Heart Association; and personal fees from Chiesi Pharmaceuticals outside the submitted work. Dr Borlaug has received research support from the 10.13039/100000002National Institutes of Health (R01 HL128526, R01 HL162828, and U01 HL160226) and the 10.13039/100000005United States Department of Defense (W81XWH2210245); has received research grant funding from 10.13039/100004325AstraZeneca, Axon, 10.13039/100004330GlaxoSmithKline, 10.13039/100004374Medtronic, Mesoblast, 10.13039/100015758Novo Nordisk, and 10.13039/100016949Tenax Therapeutics; has served as a consultant for Actelion, Amgen, Aria, BD, Boehringer Ingelheim, Cytokinetics, Edwards Lifesciences, Eli Lilly, Janssen, Merck, and Novo Nordisk; and is a named inventor (US Patent no. 10307179) for the tools and approach for a minimally invasive pericardial modification procedure to treat heart failure. Dr Cikes has received research grants and clinical study contracts to institution from 10.13039/100004336Novartis, 10.13039/100000046Abbott, 10.13039/100004319Pfizer, and CorVia; and personal fees and nonfinancial support from 10.13039/100004319Pfizer, 10.13039/100004326Bayer, 10.13039/100001003Boehringer Ingelheim, 10.13039/100004325AstraZeneca, 10.13039/100004336Novartis, Swixx, 10.13039/100020297Abiomed, 10.13039/100015362Amicus, 10.13039/100002429Amgen, 10.13039/100015758Novo Nordisk, 10.13039/100004374Medtronic, 10.13039/100006775GE Healthcare, 10.13039/100006259Teva Pharmaceutical Industries, and Krka Pharma. Dr Docherty reports that his employer, the University of Glasgow, has been remunerated by AstraZeneca for work relating to clinical trials; he has received speaker honoraria from AstraZeneca, Pharmacosmos, and Radcliffe Cardiology; has served on an advisory board for Us2.ai and Bayer AG; has served on a clinical end point committee for Bayer AG; and has received grant support from 10.13039/100001003Boehringer Ingelheim, 10.13039/100016545Roche Diagnostics, and 10.13039/100004325AstraZeneca (paid to his institution). Dr Pandey is supported by the Texas Health Resources Clinical Scholarship, the 10.13039/100005564Gilead Sciences Research Scholars Program, the 10.13039/100000049National Institute on Aging GEMSSTAR Grant (1R03AG067960-01) and Applied Therapeutics; has served on the advisory board for Roche Diagnostics; and has received nonfinancial support from 10.13039/100004319Pfizer and 10.13039/100004334Merck. Dr Kahles has served as a consultant to Bayer and Novo Nordisk and has served as a speaker for Novo Nordisk. Dr Lam is supported by a Clinician Scientist Award from the 10.13039/501100001349National Medical Research Council of Singapore; has received research support from 10.13039/100004326Bayer and 10.13039/100016545Roche Diagnostics; has served as consultant or on the Advisory Board, Steering Committee, or Executive Committee for Actelion, Alleviant Medical, Allysta Pharma, Amgen, AnaCardio AB, Applied Therapeutics, AstraZeneca, Bayer, Boehringer Ingelheim, Boston Scientific, Cytokinetics, Darma Inc, EchoNous Inc, Eli Lilly, Impulse Dynamics, Ionis Pharmaceutical, Janssen Research & Development LLC, Medscape/WebMD Global LLC, Merck, Novartis, Novo Nordisk, Prosciento Inc, Radcliffe Group Ltd, Roche Diagnostics, Sanofi, Siemens Healthcare Diagnostics and Us2.ai; and has served as cofounder and non–executive director of Us2.ai. The employer of AAV has received consultancy fees and research support from AnaCardio, 10.13039/100004325AstraZeneca, 10.13039/100002491BMS, 10.13039/100004326Bayer, 10.13039/100001003Boehringer Ingelheim, Corteria, EliLilly, 10.13039/100004334Merck, 10.13039/100019533Moderna, 10.13039/100004336Novartis, 10.13039/100015758Novo Nordisk, 10.13039/100016545Roche Diagnostics. Dr Hernandez has received grants from Intellia Therapeutics; compensation from Prolaio for consultant services; grants from 10.13039/100004326Bayer; compensation from Eli Lilly and Company for consultant services; compensation from Novo Nordisk for consultant services; employment by Duke Clinical Research Institute; compensation from Intercept Pharmaceuticals, Inc, for data and safety monitoring services; grants from 10.13039/100001003Boehringer Ingelheim; compensation from CSL Behring for consultant services; compensation from Boehringer Ingelheim for consultant services; compensation from Amgen for consultant services; compensation from Novartis for consultant services; grants from 10.13039/100018044Verily; compensation from Merck for consultant services; grants from 10.13039/100016473American Regent; compensation from AstraZeneca for consultant services; compensation from Eidos for data and safety monitoring services; compensation from Cytokinetics for consultant services; grants from 10.13039/100015758Novo Nordisk Inc, 10.13039/100004336Novartis, AstraZeneca, and 10.13039/100001238Merck; compensation from Bayer for consultant services; and compensation from Boston Scientific Corporation and Intellia Therapeutics for consultant services. Dr Lincoff has received Esperion research funding for this trial; grants from Eli Lilly, 10.13039/100006483AbbVie, 10.13039/100008322CSL, 10.13039/100004325AstraZeneca, and 10.13039/100004336Novartis; and personal fees from Novo Nordisk, Glaxo, Akebia, Endologix, Fibrogen, Provention, and Becton Dickson. Dr Petrie has received grants from 10.13039/100001003Boehringer Ingelheim, 10.13039/100004337Roche, SQ Innovations, Astra Zeneca, 10.13039/100004336Novartis, 10.13039/100015758Novo Nordisk, 10.13039/100004374Medtronic, 10.13039/100008497Boston Scientific, Horizon, Pharmacosmos; consulting fees from Boehringer Ingelheim, Novartis, AstraZeneca, Novo Nordisk, Abbvie, Bayer, Takeda, Corvia, Cardiorentis, Pharmacosmos, Siemens, and Vifor; lecture fees from Boehringer Ingelheim, Novartis, Astra Zeneca, Novo Nordisk, Abbvie, Bayer, Takeda, Corvia, Cardiorentis, Pharmacosmos, Siemens, and Vifor; board participation for Teikoku and Astra Zeneca; and is the Director of Global Clinical Trial Partners Ltd. Dr Ridker has received institutional research grant support from the 10.13039/100000050NHLBI, 10.13039/100004336Novartis, and 10.13039/100015758Novo Nordisk (to evaluate the role of anti-inflammatory agents including methotrexate, interleukin-1 inhibitors, and interleukin-6 inhibitors) as well as Kowa, Amarin, Pfizer, and Esperion; has served as a consultant to Novartis, Novo Nordisk, Janssen, Flame, Agepha, Ardelyx, Zomagen, Horizon Therapeutics, CSL Behring, and Cardio Therapeutics (entities developing anti-inflammatory therapies including as examples colchicine, interleukin-1 inhibitors, interleukin-6 inhibitors, and agents that potentially target or interact with the NLRP3 inflammasome); has additionally served as a consultant to AstraZeneca, Civi Biopharm, Glaxo Smith Kline, SOCAR, Health Outlook, Montai Health, Eli Lilly, New Amsterdam, Boehringer-Ingelheim, RTI, and Cytokinetics; has minority shareholder equity positions in Uppton, Bitteroot Bio, and Angiowave; and has received compensation for service on the Peter Munk Advisory Board (University of Toronto), the Leducq Foundation, Paris FR, and the Baim Institute (Boston, MA). Dr Fudim was supported by the 10.13039/100000968American Heart Association (20IPA35310955), Doris Duke, 10.13039/100004326Bayer, Bodyport and Verily; has received consulting fees from Abbott, Ajax, Alio Health, Alleviant, Audicor, Axon Therapies, Bayer, BodyGuide, Bodyport, Boston Scientific, Broadview, Cadence, Cardionomics, Coridea, CVRx, Daxor, Deerfield Catalyst, Edwards Lifesciences, EKO, Feldschuh Foundation, FIRE1, Gradient, Hatteras, Impulse Dynamics, InterShunt, Medtronic, NI Medical, NXT Biomedical, Pharmacosmos, PreHealth, ReCor, Shifamed, Splendo, Summacor, SyMap, Verily, Vironix, VisCardia and Zoll outside the submitted work. All other authors have reported that they have no relationships relevant to the contents of this paper to disclose.
