# Supplementary material for: Interleukin-6 and Cardiovascular Events in Healthy Adults: MESA
Source: JACC Adv. 2024 Jul 9;3(8):101063. doi: 10.1016/j.jacadv.2024.101063 (PMC11284704; doi:10.1016/j.jacadv.2024.101063)
Supplement: Supplemental Data [file mmc1.docx]

**SUPPLEMENTAL TABLE 1:** Incidence rate per 1000 person-years and cox proportional hazard models comparing the risk of outcomes in patients in tercile 1 and terciles 2 and 3.

| Tercile groups | Number of events | Person-time years | Incidence rate per 100,000 person-years | HR (95% CI); p-value | HR (95% CI); p-value |
| --- | --- | --- | --- | --- | --- |
|  |  |  |  | Model 1^*^ | Model 2^****^ |
| All-cause mortality | | | | | |
| IL6-tercile 1 | 226 | 30270 | 7.47 (6.55 to 8.51) | Ref | Ref |
| IL6-tercile 2 | 412 | 28671 | 14.4 (13.0 to 15.8) | 1.96 (1.66 to 2.30); <0.001 | 1.31 (1.08 to 1.58); p = 0.006 |
| IL6-tercile 3 | 605 | 27005 | 22.4 (20.7 to 24.3) | 3.11 (2.67 to 3.63); <0.001 | 1.86 (1.54 to 2.25); <0.001 |
| IL-6 as continuous variable |  |  |  | 1.26 (1.22 to 1.30); p < 0.001 | 1.16 (1.11 to 1.22); p < 0.001 |
| CV mortality | | | | | |
| IL6-tercile 1 | 48 | 26627.7 | 1.84 (1.39 to 2.43) | Ref | Ref |
| IL6-tercile 2 | 89 | 25976.7 | 3.43 (2.78 to 4.22) | 1.87 (1.32 to 2.65); p < 0.001 | 1.06 (0.71 to 1.58); p = 0.78 |
| IL6-tercile 3 | 157 | 25130.0 | 6.25 (5.34 to 7.31) | 3.43 (2.49 to 4.73); p < 0.001 | 1.55 (1.05 to 2.30); p = 0.029 |
| IL-6 as continuous variable |  |  |  | 1.31 (1.24 to 1.40); p < 0.001 | 1.17 (1.07 to 1.29); p = 0.001 |
| Non-CV mortality | | | | | |
| IL6-tercile 1 | 164 | 29648.1 | 5.53 (4.75 to 6.45) | Ref | Ref |
| IL6-tercile 2 | 300 | 27660.6 | 10.8 (9.69 to 12.1) | 1.99 (1.64 to 2.40); p < 0.001 | 1.43 (1.14 to 1.79); p < 0.001 |
| IL6-tercile 3 | 442 | 25663.7 | 17.2 (15.7 to 18.9) | 3.20 (2.68 to 3.83); <0.001 | 2.05 (1.65 to 2.56); p < 0.001 |
| IL-6 as continuous variable |  |  |  | 1.26 (1.22 to 1.31); p < 0.001 | 1.17 (1.11 to 1.23); p < 0.001 |
| Incident heart failure*** | | | | | |
| IL6-tercile 1 | 58 | 28799 | 2.01 (1.56 to 2.61) | Ref | Ref |
| IL6-tercile 2 | 123 | 26865 | 4.58 (3.84 to 5.46) | 2.29 (1.68 to 3.13); <0.001 | 1.43 (0.98 to 2.1); p = 0.065 |
| IL6-tercile 3 | 149 | 25037 | 5.95 (5.07 to 6.99) | 2.99 (2.21 to 4.05); <0.001 | 1.48 (0.99 to 2.19); p = 0.054 |
| IL-6 as continuous variable |  |  |  | 1.27 (1.20 to 1.34); p < 0.001 | 1.11 (1.01 to 1.22); p = 0.024 |

*Model 1 was crude (unadjusted)

**Model 2 adjusted for age at baseline, sex, race/ethnicity, current smoking, family history of CHD, waist circumference, systolic blood pressure, total cholesterol, HDL cholesterol, LDL cholesterol, diabetes, hypertension, aspirin use, anti-hypertensive medication use, insulin use, statin use, troponin T, and NT-proBNP (all assessed at baseline). .

Abbreviations: CI = confidence interval; CVD = Cardiovascular disease; HDL = high density lipoprotein; HR = hazard ratio; LDL = low density lipoprotein.

**SUPPLEMENTAL TABLE 2**: Association between incident heart failure and IL-6 levels using a competing risk analysis.

| Incident Heart Failure | | | | |
| --- | --- | --- | --- | --- |
|  | Crude Analysis | | Competing risk analysis using all-cause mortality | |
| Tercile groups | HR (95% CI); p-value | HR (95% CI); p-value | HR (95% CI); p-value | HR (95% CI); p-value |
|  | Model 1^*^ | Model 2^****^ | Model 1^*^ | Model 2^****^ |
| IL6-tercile 1 | Ref | Ref | Ref | Ref |
| IL6-tercile 2 | 2.29 (1.68 to 3.13); <0.001 | 1.43 (0.98 to 2.1); p = 0.065 | 2.18 (1.59 to 2.98); p < 0.001 | 1.39 (0.92 to 2.11); p = 0.12 |
| IL6-tercile 3 | 2.99 (2.21 to 4.05); <0.001 | 1.48 (0.99 to 2.19); p = 0.054 | 1.64 (1.41 to 1.91); p < 0.001 | 1.10 (0.90 to 1.36); p = 0.33 |
| IL-6 as continuous variable | 1.27 (1.20 to 1.34); p < 0.001 | 1.11 (1.01 to 1.22); p = 0.024 | 1.24 (1.18 to 1.31); p < 0.001 | 1.08 (0.99 to 1.18); p = 0.085 |

*Model 1 was crude (unadjusted)

**Model 2 adjusted for age at baseline, sex, race/ethnicity, current smoking, family history of CHD, waist circumference, systolic blood pressure, total cholesterol, HDL cholesterol, LDL cholesterol, diabetes, hypertension, aspirin use, anti-hypertensive medication use, insulin use, statin use, troponin T, and NT-proBNP (all assessed at baseline).
